# A new hybrid optimization intelligence for cable-driven continuum robot motion: two-obstacle simulation and real-data assessment

**DOI:** 10.3389/frobt.2026.1935721

**Published:** 2026-09-30

**Authors:** Selman Djeffal, Abdelhamid Ghoul, Mohamed Razi Morakchi, Badreddine Bendriss, Merabet Eddine Salah, Atef Chibani

**Affiliations:** 1 Department of Mechanical Engineering, National Polytechnic School of Constantine, Constantine, Algeria; 2 Automatic Control, Department of Computer Science, Electrical and Space Engineering, Luleå University of Technology, Luleå, Sweden; 3 Department of Electromechanics, Faculty of Construction Engineering, Mouloud Mammeri University of Tizi Ouzou, Tizi Ouzou, Algeria; 4 Department of Electrotechnics, Setif 1 Ferhat Abbas University, Sétif, Algeria; 5 Department of Electrotechnics, Mentouri Brothers, Constantine 1, Constantine, Algeria; 6 Research Center in Industrial Technologies (CRTI), Algiers, Algeria

**Keywords:** cable-driven robot, continuum robot, differential evolution, forward kinematics, Harris hawks optimization, hybrid optimization, inverse kinematics, obstacle avoidance

## Abstract

**Introduction:**

Cable-driven continuum robots enable dexterous motion in confined spaces, but inverse kinematics remains difficult because different internal shapes and cable states can produce the same end-effector position.

**Methods:**

This study presents a design-aware hybrid optimization framework, DA-IHHO-DELS, for inverse kinematics of a three-section continuum robot. A piecewise-constant-curvature forward model generates physically consistent section positions, cable-length estimates, and feasibility terms. The optimizer combines geometry-consistent seeding, opposition learning, improved Harris hawks search, differential-evolution exploitation, grey-wolf leader guidance, Cauchy/Levy perturbations, and bounded local refinement. Its multi-objective score includes the arc-length consistency residual, orientation error, cable-length dispersion, inter-section smoothness, actuation effort, and constraint violation, and it is compared with GA, ABC, ACO, DE, HHO, TLBO, BOA, GWO, WOA, and POA.

**Results:**

In the regenerated obstacle-constrained simulation, DA-IHHO-DELS achieved a median $F_{1}$ residual of 0.00491 mm, a median reconstructed Cartesian error of 0.00255 mm, 100% success under the strict $F_{1}<0.01$ criterion, the lowest median aggregate score, and a mean runtime of 0.56883 s. A secondary benchmark using measured CBHA/Robotino XT actuator lengths further assessed transfer of the geometry-guided optimization consistency to real measured continuum-robot data.

**Discussion:**

The results show that design-aware hybrid optimization can combine analytical continuum-robot kinematics with robust search to obtain accurate, feasible, and smooth inverse-kinematic solutions. The real-data benchmark supports consistency transfer while remaining distinct from a direct hardware test of the same three-section cable-driven robot.

## 1 Introduction

Continuum robots are formed by continuously deformable backbones rather than discrete rigid links. Their body can bend along smooth arcs, enabling motion through cluttered, narrow, and contact-rich environments where classical manipulators are limited. Cable-driven continuum robots are especially attractive because actuation can remain at the base while tendon or cable routing produces distributed bending along the body. This design improves compactness and dexterity, but it also creates a difficult kinematic problem: the actuator variables, internal section curvatures, and end-effector pose are coupled by nonlinear geometry.

In Webster and Jones (2010), the authors established a widely used review of constant-curvature and piecewise-constant-curvature formulations for continuum manipulators. In Burgner-Kahrs et al. (2015), the authors examined design and kinematic challenges in continuum mechanisms and showed why section-wise curvature assumptions remain useful for practical control. In Walker (2013), continuum-robot fundamentals were connected to bio-inspired manipulation, while Trivedi et al. (2008) compared soft-robot modeling requirements with biological actuation principles. Along the same line, Rus and Tolley (2015) highlighted the need for unified design, fabrication, and control strategies in soft robots, whereas Thuruthel et al. (2018) reviewed closed-loop control strategies and identified model uncertainty as a central obstacle.

From a kinematic viewpoint, Jones and Walker (2006) developed multisection continuum-robot kinematics using serial constant-curvature transformations. In comparison, Godage and Walker (2011) investigated modal representations for multisection arms and showed how compact coordinates can reduce complexity. Meanwhile, Rucker et al. (2010) introduced a geometrically exact continuum model suitable for externally loaded structures, whereas Renda et al. (2012) proposed a three-dimensional tendon-driven soft-manipulator model inspired by the octopus arm. Similarly, Boyer et al. (2006) addressed continuous-body modeling from a dynamic-control perspective, while Mahapatra et al. (2020) investigated generally routed cable-driven robots and emphasized the impact of cable paths and disk layout on accuracy.

Several recent studies have focused directly on tendon- and cable-driven robot modeling. In Rao et al. (2021), the authors benchmarked tendon-driven continuum-robot models and demonstrated that the chosen physical assumptions strongly influence prediction quality. In Bhalkikar et al. (2024), multiple-segment cable routing and varying cable offsets were analyzed, which is closely related to the discrete cable representation considered here. In Barrientos-Diez et al. (2021), real-time continuum-robot kinematics were modeled and validated experimentally, confirming the importance of computationally efficient inverse kinematics. In Chikhaoui et al. (2019), embedded actuation and kinematic performance were investigated, while Shi et al. (2017) surveyed shape-sensing techniques that can complement cable-length based control. More broadly, Dupont et al. (2021) summarized the progress of medical robotics, where continuum manipulators are increasingly relevant because of their compliance and small operational footprint.

More recently, Bhalkikar et al. (2026) developed forward and inverse kinetostatic models for a cable-driven continuum robot with varying radial cable offsets. Their formulation explicitly considers load-dependent backbone equilibrium and shows how cable routing, external loading, and cable-disk friction can alter the predicted profile. This work complements the present study: here, DA-IHHO-DELS is formulated around a fast PCC inverse-kinematic model, whereas the kinetostatic formulation provides a higher-fidelity route when force equilibrium and non-ideal transmission effects must be resolved.

Optimization and learning have also been used to compensate for the limitations of purely analytical models. In Thuruthel et al. (2019), model-based reinforcement learning was used for closed-loop soft-manipulator control. In Wang et al. (2016), learning from demonstration supported motion planning for flexible soft robots. In Djeffal et al. (2024), deep reinforcement learning was applied to a spatial three-section continuum robot, showing the value of data-supported control. More recently, Djeffal et al. (2026) introduced a geometrically consistent discrete mapping for multisection continuum-robot motion and validated learning-based inverse kinematics on a Robotino® XT/CBHA platform, highlighting the importance of deterministic actuation labels, experimental benchmarking, and low-latency inference. That study motivates the present emphasis on geometric consistency, while the contribution here is different: the inverse-kinematic map is solved through a constrained multi-objective metaheuristic search with explicit obstacle handling rather than through DNN, INP, or GPR regression. In Escande et al. (2012), experimental cable-length and RobotinoXT validation demonstrated the importance of comparing generated actuation commands with real measured cable behavior. In Djeffal and Mahfoudi (2023), PSO and TLBO were compared with other metaheuristics for multisection continuum-robot inverse kinematics; compared with that work, the present study strengthens the optimizer by combining design-aware initialization, HHO exploration, DE exploitation, leader guidance, and local refinement.

The optimization baseline is intentionally broad. In Holland (1992), genetic algorithms were formalized as population-based adaptive search. In Karaboga (2005), the artificial bee colony principle was introduced for numerical optimization, while Dorigo et al. (1996) formulated ant-colony cooperation. In Storn and Price (1997), differential evolution was proposed as a simple but powerful continuous optimizer. In Heidari et al. (2019), Harris hawks optimization introduced escaping-energy behavior, whereas Rao et al. (2011) proposed teaching-learning-based optimization. In Arora and Singh (2019), butterfly optimization used fragrance-inspired search; Mirjalili et al. (2014) proposed grey-wolf hierarchy; Mirjalili and Lewis (2016) introduced whale-spiral exploitation; and Trojovska and Dehghani (2022) described pelican optimization. These separate search philosophies provide a balanced reference set for evaluating whether the proposed hybrid strategy truly improves inverse-kinematic accuracy.

This work addresses the above limitations by combining a deterministic forward/inverse geometric formulation with a hybrid design-aware optimizer. The main contribution is DA-IHHO-DELS, a proposed inverse-kinematic method that improves the search behavior of HHO through design-aware seeding, opposition learning, DE mutation, GWO-style leader guidance, random long jumps, and bounded local refinement. The approach is evaluated against ten widely used algorithms: genetic algorithm (GA), artificial bee colony (ABC), ant colony optimization (ACO), differential evolution (DE), Harris hawks optimization (HHO), teaching-learning-based optimization (TLBO), butterfly optimization algorithm (BOA), grey wolf optimizer (GWO), whale optimization algorithm (WOA), and pelican optimization algorithm (POA) (Holland, 1992; Karaboga, 2005; Dorigo et al., 1996; Storn and Price, 1997; Heidari et al., 2019; Rao et al., 2011; Arora and Singh, 2019; Mirjalili et al., 2014; Mirjalili and Lewis, 2016; Trojovska and Dehghani, 2022).

The remainder of this paper is organized as follows. Section 2 introduces the forward and inverse kinematic formulation of the three-section cable-driven continuum robot. Section 3 details the proposed DA-IHHO-DELS optimization strategy and its multi-objective formulation. Section 4 describes the simulation and experimental protocols. Section 5 discusses the numerical and experimental results. Finally, Section 6 concludes the paper and outlines future work.

## 2 Forward and inverse kinematics of the three-section continuum robot

### 2.1 Forward kinematic model

The robot is modeled as three serial continuum sections. Each section is represented by a constant-curvature arc of length $L_{j}$ , bending angle $\theta_{j}$ , and bending-plane orientation $\phi_{j}$ . For one section, the local rotation is

$$R_{j-1}^{j}=R_{z}\left(\phi_{j}\right)\;R_{y}\left(\theta_{j}\right)\;R_{z}\left(-\phi_{j}\right),$$ (1)

and the arc endpoint translation is

$$p_{j-1}^{j}=\left\{\begin{array}{cc} {\left[0,0,L_{j}\right]}^{T}, & |\theta_{j}|<\epsilon ,\\ R_{z}\left(\phi_{j}\right){\left[\rho_{j}\left(1-\cos\theta_{j}\right),0,\rho_{j}\sin\theta_{j}\right]}^{T}, & \text{otherwise}, \end{array}\right.\;\rho_{j}=\frac{L_{j}}{\theta_{j}}.$$ (2)

The homogeneous transformation of section j is therefore

$$T_{j-1}^{j}=\left[\begin{array}{cc} R_{j-1}^{j} & p_{j-1}^{j}\\ 0_{1\times 3} & 1 \end{array}\right],$$ (3)

and the global end-effector transformation for the three-section robot is

$$T_{0}^{3}=T_{0}^{1}T_{1}^{2}T_{2}^{3},\;x=T_{0}^{3}{\left[\begin{array}{cccc} 0 & 0 & 0 & 1 \end{array}\right]}^{T}.$$ (4)

Figure 1 summarizes the section-level geometric convention, where each conical section is described by the bending angle $\theta_{j}$ , bending-plane orientation $\phi_{j}$ , disk radius $r_{j}$ , and cable angular locations. Figure 2 illustrates the complete mapping from cable-length actuation to configuration and task spaces, including the forward-kinematic, inverse-kinematic, optimization, and validation stages.

**FIGURE 1:**
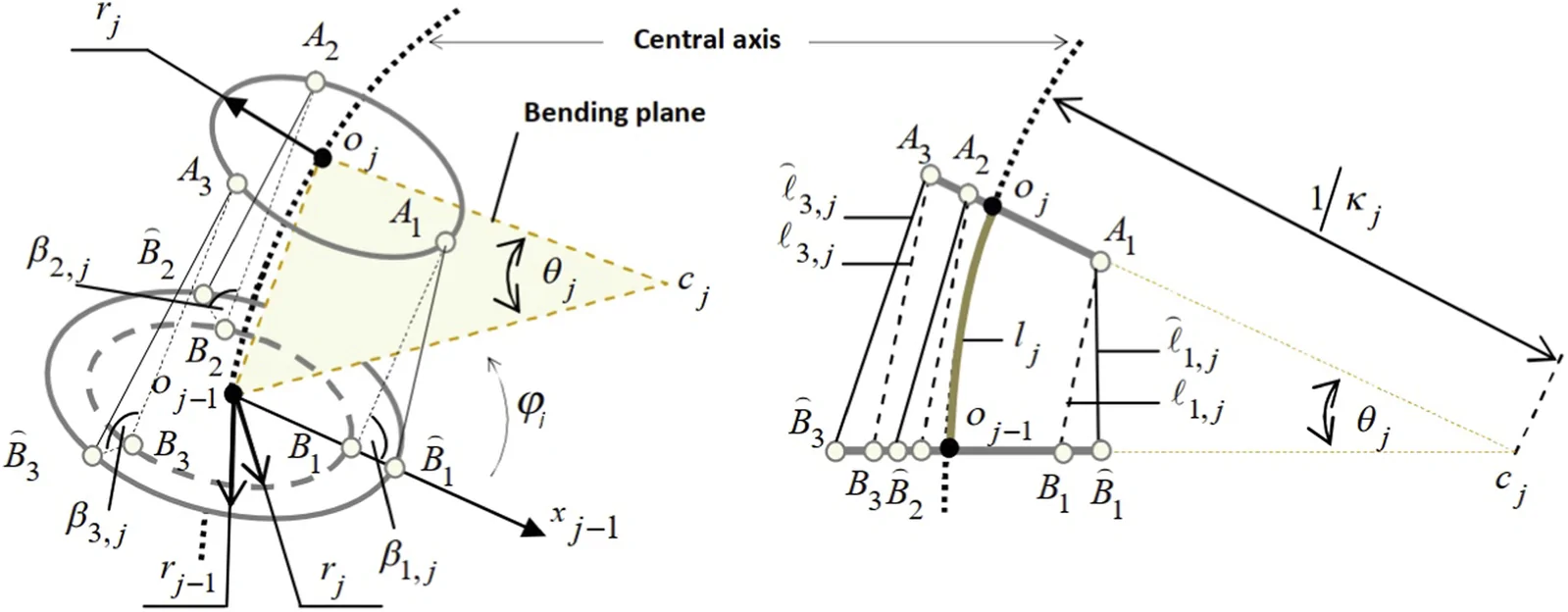
Geometric model of a conical cable-driven continuum section.

**FIGURE 2:**
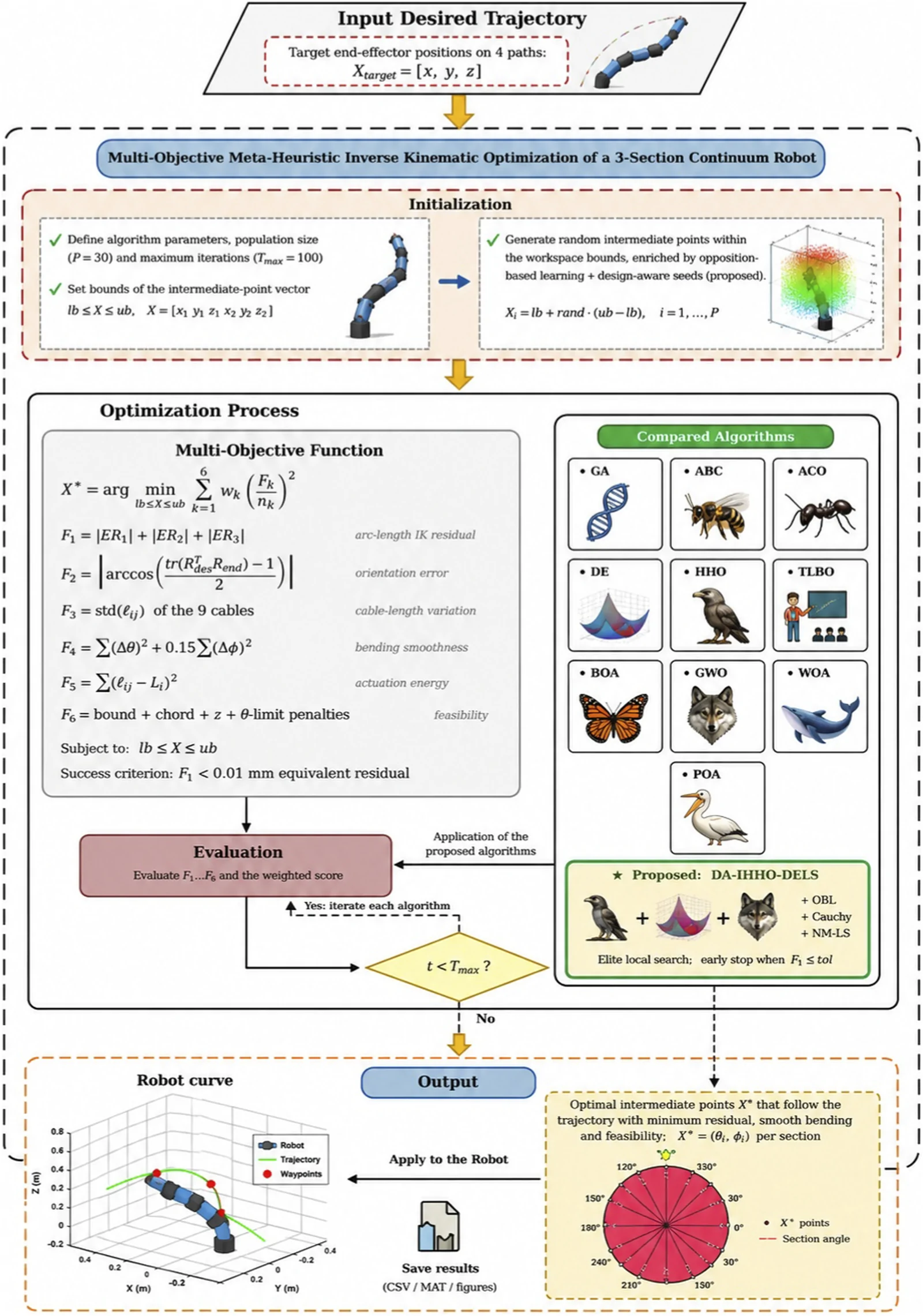
Methodological workflow of the continuum-robot inverse-kinematics framework.

The forward kinematic model in Equation 4 was further used to construct the three-dimensional reachable workspace shown in Figure 3. The admissible bending angles and bending-plane orientations of the three sections were densely sampled, and the corresponding end-effector position was calculated from the serial PCC transformations. Consequently, each point in the workspace cloud represents an end-effector position that can be attained by at least one sampled robot configuration under the prescribed geometric limits. Four representative robot configurations are superimposed to illustrate the large shape redundancy associated with different combinations of section curvatures and bending-plane orientations.

**FIGURE 3:**
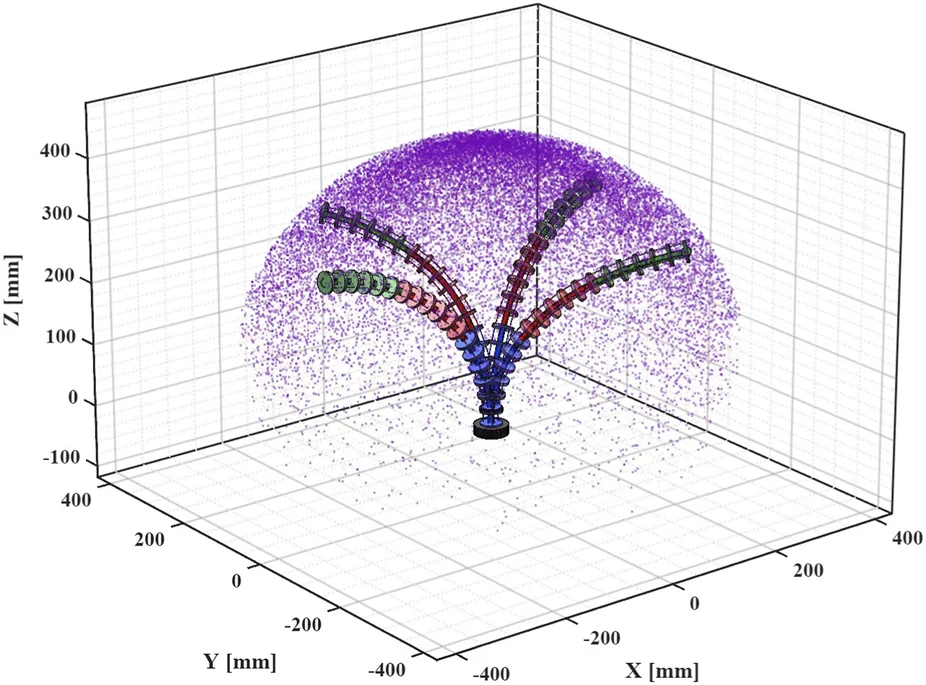
Forward-kinematic reachable workspace configurations of the three-section continuum robot.

The same forward-kinematic samples were also evaluated for kinematic singularity using a numerical 6×6 pose Jacobian containing both translational and rotational differential components. Near-singular configurations were identified from the normalized singular-value condition $\sigma_{\min}\left(J\right)/\sigma_{\max}\left(J\right)<10^{-2}$ and are highlighted separately in Figure 3. These regions correspond to configurations in which the Jacobian becomes strongly ill-conditioned and the robot loses local Cartesian dexterity in one or more motion directions. They therefore identify portions of the otherwise reachable workspace where small task-space motions may require disproportionately large changes in the section variables, making such configurations less desirable for accurate trajectory tracking and motion planning.

The PCC model is used here as a quasi-static kinematic approximation. It is most appropriate when each section bends approximately as a circular arc and distributed loading is modest. Gravity, external tip/contact forces, cable-disk friction, backbone shear/extension, and material hysteresis can produce non-constant curvature and path-dependent actuation, so the present formulation should not be interpreted as a full kinetostatic or dynamic model. These effects are expected to increase model mismatch as loading and deformation become more severe, and they are treated as limitations of the present study rather than being absorbed implicitly into the optimizer.

### 2.2 Discrete disk and cable-length representation

The discrete representation divides each section into m spacer-disk intervals of length $L_{\text{seg}}=L_{j}/m$ . The position of cable c on disk k is

$$v_{k,c}^{\text{local}}={\left[\begin{array}{cccc} r_{k}\cos\gamma_{c} & r_{k}\sin\gamma_{c} & 0 & 1 \end{array}\right]}^{T},\;\gamma_{c}=\gamma_{0}+\frac{2\pi \left(c-1\right)}{n_{c}},$$ (5)

where $r_{k}$ is the disk-hole radius and $n_{c}$ is the number of cables per section. The global cable point is

$$V_{k,c}=T_{0}^{k}v_{k,c}^{\text{local}},$$ (6)

and the corresponding cable length is computed by summing the Euclidean distances between consecutive disk points:

$$L_{c}=\overset{m-1}{\underset{k=1}{\sum }}{\left\|V_{k+1,c}-V_{k,c}\right\|}_{2}.$$ (7)

This discrete cable-length formulation follows the same physical idea as experimental cable-length validation: the measured actuator state should correspond to a unique and reproducible geometric backbone state.

### 2.3 Inverse kinematic parameter extraction

The inverse formulation used in the code represents the decision vector as the two internal section-junction points

$$q_{\text{IK}}={\left[x_{1},y_{1},z_{1},x_{2},y_{2},z_{2}\right]}^{T}.$$ (8)

For the first section, the bending variables are recovered from the first junction point as

$$\phi_{1}=atan2\left(y_{1},x_{1}\right),\;\theta_{1}=2\;atan2\left(\sqrt{x_{1}^{2}+y_{1}^{2}},z_{1}+\epsilon \right).$$ (9)

For the second and third sections, the relative segment vectors are transformed into the corresponding local frames, after which the same relation as Equation 9 is applied. The arc-length consistency residual is explicitly defined in Equation 10.

$$E_{j}=L_{j}-D_{j}\frac{\theta_{j}/2}{\sin\left(\theta_{j}/2\right)},$$ (10)

where $D_{j}$ is the chord length of the corresponding section. The original residual minimized in the study is

$$F_{1}=|E_{1}|+|E_{2}|+|E_{3}|.$$ (11)

It is important to distinguish $F_{1}$ from Cartesian tip-position error. In the adopted inverse parameterization, the desired endpoint is imposed explicitly as the third section endpoint, whereas $F_{1}$ tests whether the two optimized junction points and that fixed endpoint are geometrically compatible with the nominal section arc lengths. For reporting, the revised implementation additionally reconstructs the three-section PCC chain from the recovered $\left(\theta_{j},\phi_{j}\right)$ and nominal $L_{j}$ and evaluates

$$e_{p}={\left\|p_{\text{FKM}}\left(\theta_{1},\phi_{1},\ldots ,\theta_{3},\phi_{3};L_{1},L_{2},L_{3}\right)-p_{\text{des}}\right\|}_{2}.$$ (12)

Thus, $F_{1}$ is an arc-length closure/consistency residual, while $e_{p}$ is the reconstructed end-effector position error. A small $F_{1}$ indicates that the recovered PCC geometry is compatible with the prescribed section lengths; $e_{p}$ reports the Cartesian consequence of any remaining inconsistency. The optimizer continues to use $F_{1}$ to preserve continuity with the original formulation, and the revised MATLAB file exports $e_{p}$ as a separate diagnostic.

## 3 Proposed DA-IHHO-DELS methods

### 3.1 Multi-objective optimization problem

The proposed method solves the inverse kinematics as a bounded multi-objective optimization problem. For compact labels in the comparative figures, PM denotes the proposed method, namely DA-IHHO-DELS; the complete algorithm name is retained in the equations, tables, and discussion. Besides the primary residual $F_{1}$ in Equation 11, the score includes orientation accuracy, cable variation, inter-section smoothness, actuation effort, and feasibility penalties:

$$F_{2}=\left|\cos^{-1}\left(\frac{tr\left(R_{\text{des}}^{T}R_{\text{end}}\right)-1}{2}\right)\right|,$$ (13)

$$F_{3}=std\left(L_{\text{cable}}\right),$$ (14)

$$F_{4}={\left(\theta_{2}-\theta_{1}\right)}^{2}+{\left(\theta_{3}-\theta_{2}\right)}^{2}+0.15\left[\Delta \phi_{21}^{2}+\Delta \phi_{32}^{2}\right],$$ (15)

$$F_{5}=\underset{j,c}{\sum }{\left(L_{j,c}-L_{j}\right)}^{2},$$ (16)

$$F_{6}=P_{\text{bounds}}+P_{\text{chord}}+P_{z}+P_{\theta }+P_{\text{obs}}.$$ (17)

For each spherical obstacle k with center $o_{k}$ , radius $r_{k}$ , prescribed clearance $c_{k}$ , and robot envelope radius $r_{b}$ , collision avoidance is imposed through the sampled-backbone inequality

$$g_{k}\left(q\right)=r_{k}+r_{b}+c_{k}-\underset{p\in B\left(q\right)}{\min}{\left\|p-o_{k}\right\|}_{2}\leq 0,$$ (18)

where B(q) contains 46 PCC backbone samples per section. The obstacle penalty used in $F_{6}$ is

$$P_{\text{obs}}=\underset{k}{\sum }{\left[\frac{\max\left(0,g_{k}\right)}{\eta_{\text{obs}}}\right]}^{2},\;\eta_{\text{obs}}=10\;mm.$$ (19)

Consequently, a candidate is penalty-free only when the complete sampled backbone remains outside the obstacle radius, robot envelope, and requested safety clearance.

The scalar aggregate score is

$$J\left(q_{\text{IK}}\right)=\overset{6}{\underset{i=1}{\sum }}w_{i}{\left(\frac{F_{i}}{\eta_{i}}\right)}^{2},$$ (20)

where $w_{i}$ and $\eta_{i}$ are weighting and normalization factors. The strong weight assigned to $F_{1}$ preserves the original inverse-kinematic residual, while the remaining terms enforce physically smooth, feasible, and actuator-consistent solutions.

For reproducibility, the fixed normalization and weight vectors used for every method and every stochastic run are $\eta =\left[10^{-2},10^{-2},5,1,800,1\right]$ and w=[1.00,0.06,0.03,0.03,0.01,1.00] , respectively. The normalizers place quantities with different physical scales in comparable numerical ranges, while the weights keep the geometric closure residual and feasibility terms dominant and treat orientation, cable dispersion, inter-section smoothness, and actuation effort as secondary regularizers. These values are not changed between algorithms.

### 3.2 Hybrid search operators

DA-IHHO-DELS improves standard HHO using five complementary mechanisms. First, geometry-aware seeds are constructed from the target position and injected into the initial population. Second, opposition-based learning evaluates opposite candidates around the bounded design domain. Third, adaptive DE mutation moves individuals toward the current best while preserving population diversity. Fourth, GWO-style leader combination uses the best three solutions to stabilize exploitation. Fifth, Cauchy/Levy perturbations and bounded local search are used when the optimizer must escape a local basin or polish a near-feasible inverse-kinematic solution.

Algorithm 1 **DA-IHHO-DELS for design-aware continuum-robot inverse kinematics.**

**Require:** Target pose $\left(x_{\text{des}},R_{\text{des}}\right)$ , bounds $\left[l_{b},u_{b}\right]$ , robot parameters, maximum iterations $N_{it}$

**Ensure:** Best inverse-kinematic vector $q_{\text{IK}}^{⋆}$ and score $J^{⋆}$

1: Generate a random population P and an opposition population using Equation 8
2: Add design-aware seeds derived from the target and section lengths
3: **for** each candidate $q_{i}\in P$ **do**
4:  Decode $q_{i}$ into $\left(\theta_{j},\phi_{j}\right)$ using Equation 9
5:  Compute FKM using Equations 1–4
6:  Compute cable lengths using Equations 5–7
7:  Evaluate $F_{1},\ldots ,F_{6}$ using Equations 11–17 and J using Equation 20
8: **end for**
9: Select alpha, beta, and delta leaders from the best candidates
10: **for** t=1 to $N_{it}$ **do**
11:  **for** each candidate $q_{i}$ **do**
12:   **if** rand<0.34 **then**
13:    Apply adaptive DE current-to-best mutation and crossover
14:   **else if** rand<0.68 **then**
15:    Apply GWO leader-guided update using alpha, beta, and delta
16:   **else**
17:    Apply HHO escaping-energy update with Cauchy/Levy perturbation
18:   **end if**
19:   Bound the candidate inside $\left[l_{b},u_{b}\right]$ and evaluate Equation 20
20:   **if** the candidate improves its previous score **then**
21:    Accept the candidate
22:   **end if**
23:  **end for**
24:  **if** t is a polishing iteration **and** $F_{1}$ is above tolerance **then**
25:   Run bounded local refinement on the elite candidate using Equation 20
26:  **end if**
27:  **while** $F_{1}$ is below the deployment tolerance and $t<N_{it}$ **do**
28:   Pad the convergence history with the current best score and **break**
29:  **end while**
30: **end for**
31: **return** $q_{\text{IK}}^{⋆}$ and $J^{⋆}$

The MATLAB implementation, cable-length data, generated tables, and figure-generation scripts can be provided with the paper to reproduce the obtained results, inspect the DA-IHHO-DELS workflow, and support future improvements of the proposed method.

## 4 Simulation and real-data protocol

The numerical comparison uses one reachable three-section target case and ten independent stochastic runs per method. The same target, bound constraints, objective definition, and stopping tolerance are supplied to every optimizer. DA-IHHO-DELS additionally uses its design-aware seeding, adaptive hybrid operators, and bounded local-refinement stage. The visible trajectory contains two spherical obstacles, and multiple robot configurations are displayed along the path to illustrate the section-level bending states produced by the compared methods. Figure 4 compares the 11 optimization methods on the same red target trajectory and obstacle geometry, allowing the feasibility and visual consistency of the resulting bending configurations to be inspected under identical conditions.

**FIGURE 4:**
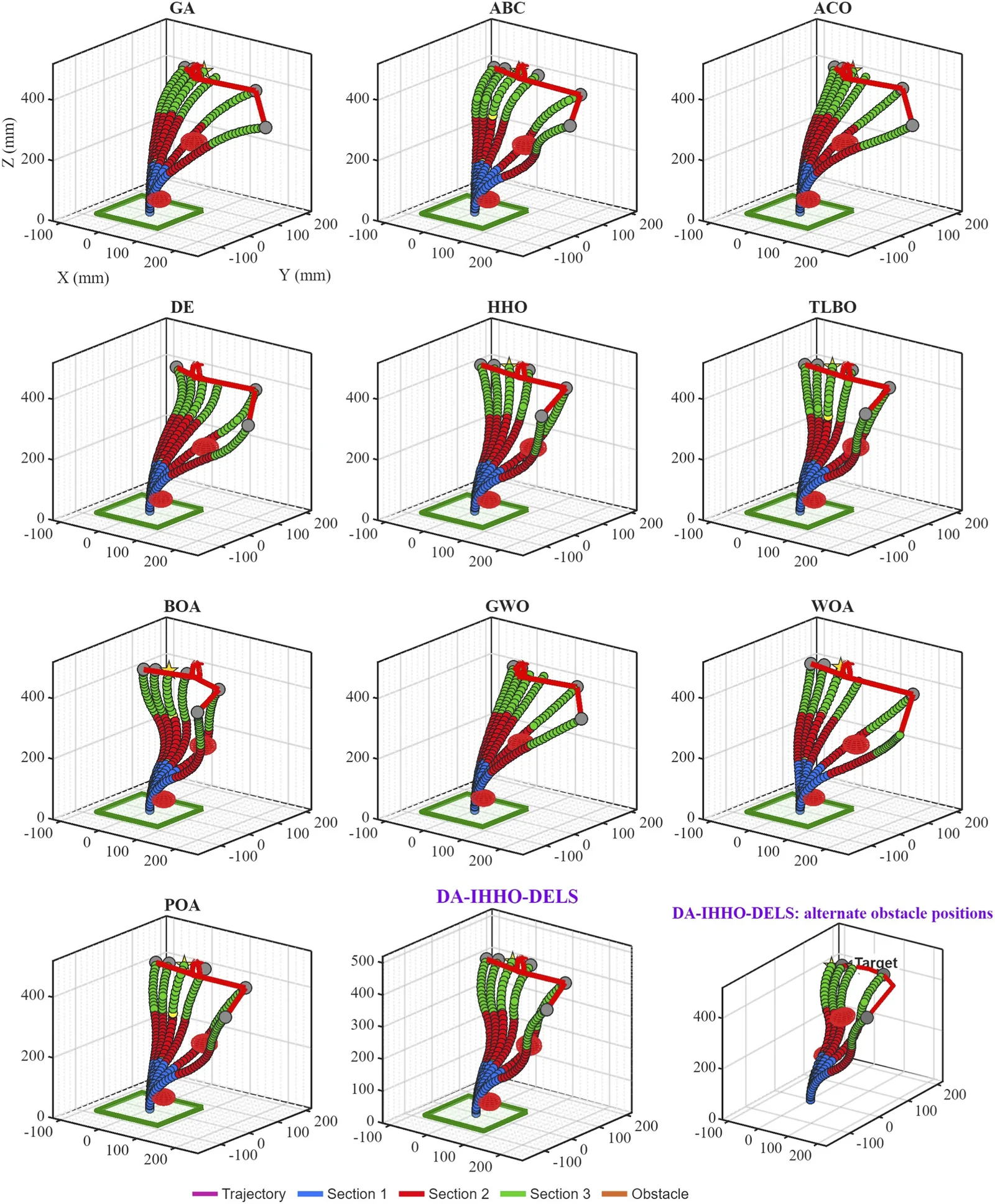
Three-section robot simulation with 11 optimization methods.

The theoretical cable-driven robot consists of three 150 mm PCC sections, giving a 450 mm nominal backbone length. Each section is discretized into eight disk intervals, the robot-envelope radius used by collision checking is 10 mm, and three cables are uniformly spaced by 120° around each section. The two obstacles have radii of 20 and 17 mm, respectively, with an 8 mm safety clearance. For obstacle k , feasibility is evaluated from the minimum sampled backbone-to-center distance and the constraint in Equations 18, 19. The same obstacle geometry and safety margin are enforced for all 11 algorithms during optimization, not only during visualization.

All stochastic optimizations were executed in MATLAB R2025a on a 64-bit Windows HP OMEN workstation equipped with a 12th-generation Intel Core i7 processor, 32 GB RAM, and an NVIDIA GeForce GTX graphics processor. The optimization code is CPU-based and does not invoke gpuArray or CUDA kernels. MATLAB tic/toc was used around every independent run. The measured mean wall-clock times were 4.6785 s (GA), 9.2969 s (ABC), 4.6048 s (ACO), 4.6380 s (DE), 4.6553 s (HHO), 9.1709 s (TLBO), 4.6788 s (BOA), 4.6439 s (GWO), 4.6037 s (WOA), 9.2385 s (POA), and 0.56883 s for PM. Thus, under the implementation and stopping rules used in this study, PM required only 6.1%–12.4% of the baseline wall-clock time. This timing comparison is reported as implementation-level deployment cost; it is not presented as an equal-function-evaluation complexity proof because the proposed method includes adaptive termination and local refinement. The theoretical results are presented through two complementary statistical views. Figure 5 presents a 3×2 stochastic distribution comparison and displays the ten-run distributions of $F_{1}$ , reconstructed Cartesian tip-position error, orientation error, cable-length variation, smoothness, and the aggregate objective J . A monotonic balanced display scaling is used only in the strongly unbalanced panels so that the small-error distributions remain visible; all numerical statistics in the tables are calculated from the original, untransformed values. PM denotes the proposed method DA-IHHO-DELS.

**FIGURE 5:**
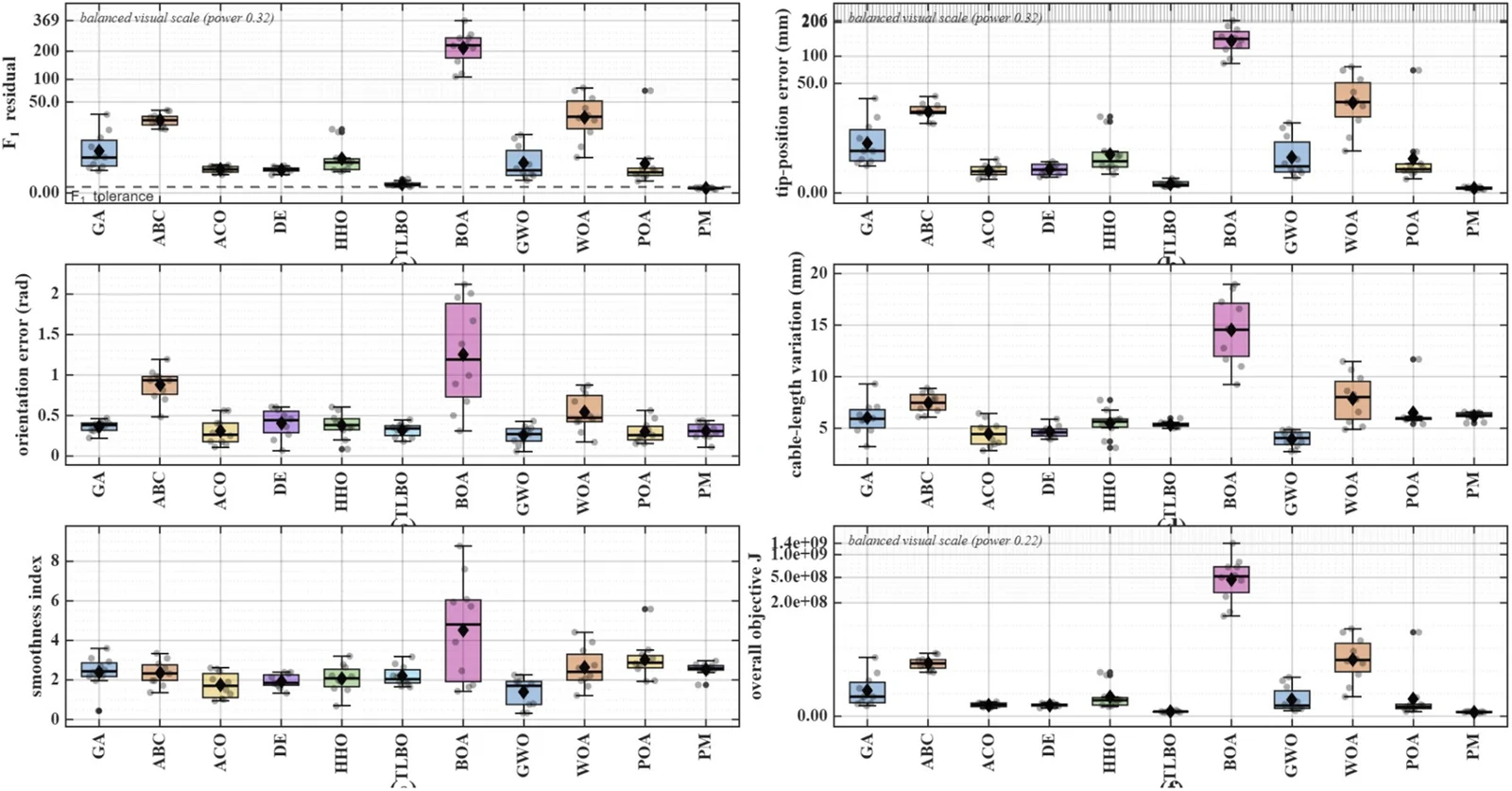
Six-metric stochastic comparison of the eleven optimization methods.

Figure 6 provides a second, nonredundant view using conventional logarithmic axes and convergence histories. The residual panel shows the strict $F_{1}<10^{-2}$ acceptance threshold, the smoothness panel retains the run-wise ordering, the aggregate-score panel exposes the several-order-of-magnitude separation between algorithms, and the convergence panel shows how quickly the median best-so-far objective stabilizes. Figure 7 then adds a geometric interpretation of these statistics by overlaying ten PM backbone configurations during obstacle-aware trajectory following. Together, Figures 5–7 make the stochastic accuracy, robustness, convergence behavior, and shape feasibility directly auditable.

**FIGURE 6:**
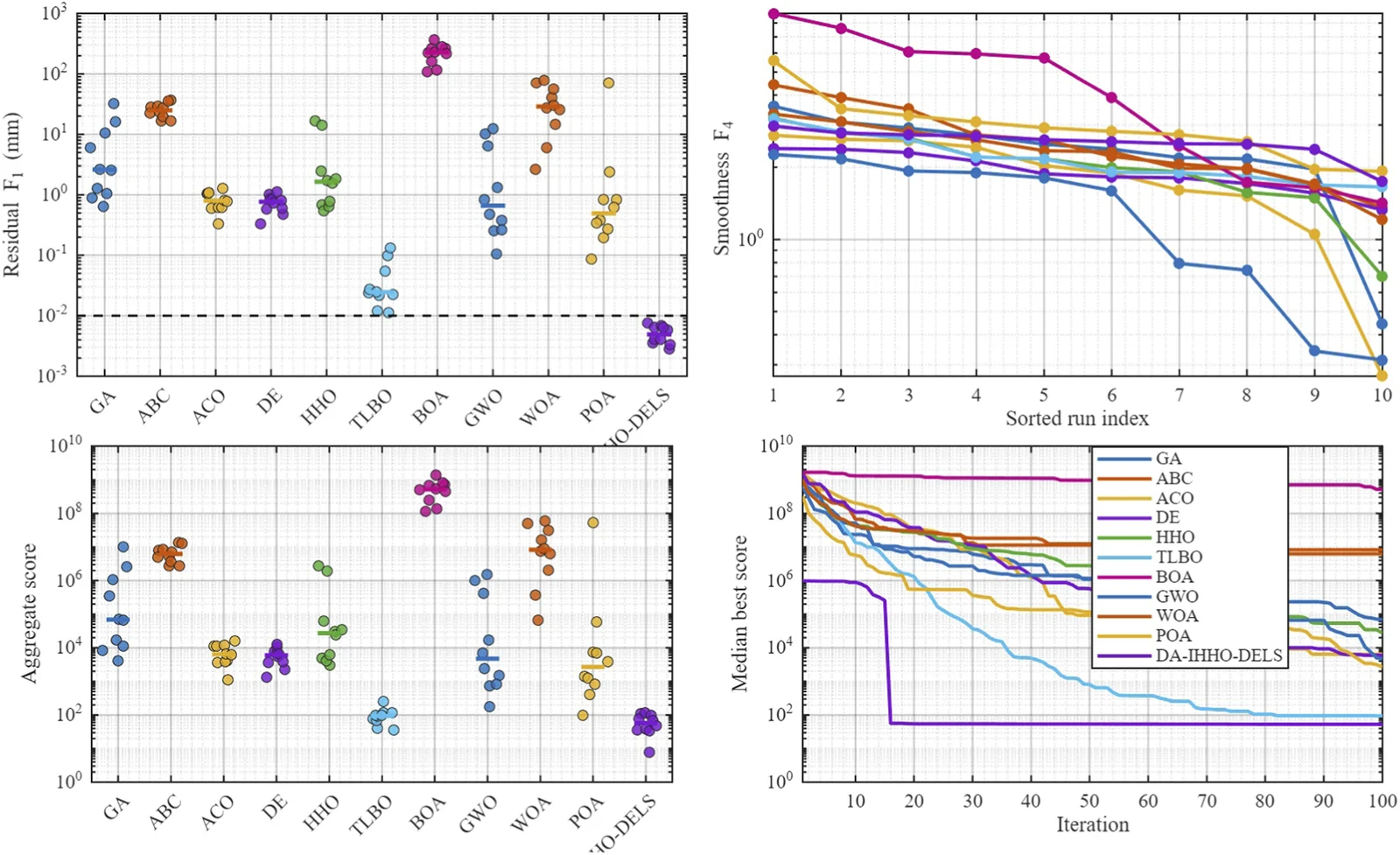
Stochastic robustness and convergence comparison.

**FIGURE 7:**
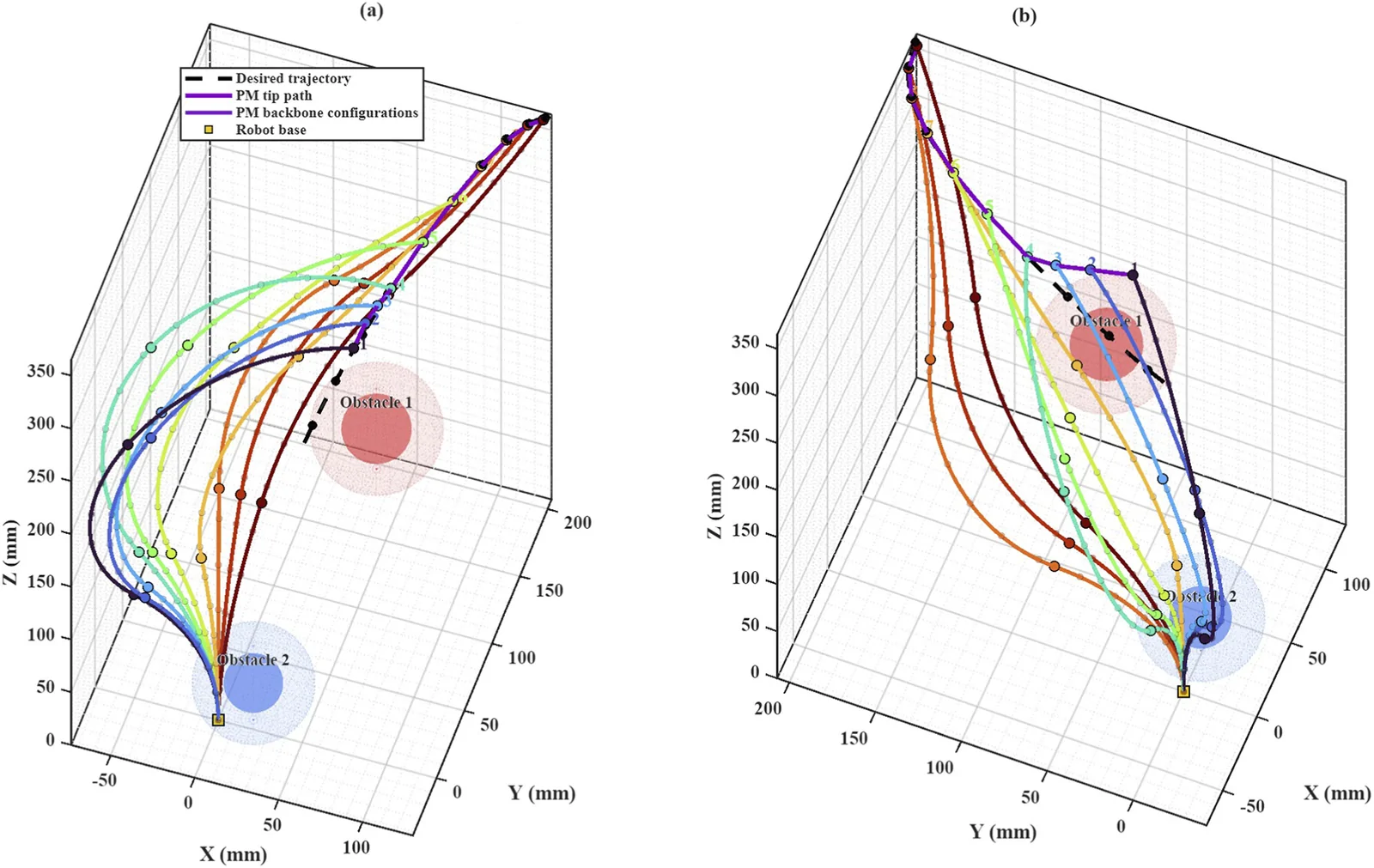
Obstacle-aware trajectory following using ten PM backbone configurations. **(a)** Perspective view of PM trajectory following. **(b)** Second view showing backbone detours around both obstacles.

Figure 7 adds a shape-level interpretation to the statistical findings. Here, PM denotes the proposed method DA-IHHO-DELS. The early PM scenarios show how the robot departs from the base region while preserving clearance from Obstacle 2, which occupies the lower workspace. The middle scenarios then sweep laterally around Obstacle 1 without abrupt posture changes, which is consistent with the favorable smoothness distribution already observed in Figures 5, 6. The final scenarios converge onto the upper target samples with the end effector still lying on the desired trajectory. This is an important practical finding because continuum-robot inverse kinematics is often judged only through endpoint error, whereas the new figure shows that PM also preserves a coherent family of feasible backbone shapes throughout the motion. The two complementary views help reveal detours that would be less obvious from a single camera angle, thereby supporting the claim that the gain of PM is structural rather than accidental.

The real-data component is a secondary benchmark based on the supplied Data_1.mat measurements associated with the Compact Bionic Handling Assistant (CBHA)/Robotino® XT platform, rather than a new experiment performed on the same three-section cable-driven robot used in the theoretical model. The experimental platform combines a Robotino mobile base with a CBHA composed of a gripper, a rotating interface, and two flexible polyamide sections. Each flexible section contains three independently controlled pneumatic tubes, and a central rod mechanically limits axial extension. Because the CBHA does not provide absolute internal pose sensing, six embedded potentiometer signals are used to measure tube elongation, while an external KUKA LBR Med manipulator is mechanically coupled to the CBHA tip to provide reference pose measurements and to generate the target motion. The reference pose is described by $\left\{X_{\mathrm{KUKA}},Y_{\mathrm{KUKA}},Z_{\mathrm{KUKA}},\Phi_{\mathrm{KUKA}},\theta_{\mathrm{KUKA}},\Psi_{\mathrm{KUKA}}\right\}$ and transformed between the measurement frames before comparison. The published experimental protocol uses a 50 Hz sampling frequency, hardware-triggered Real-Time UDP synchronization with latency below 2 ms, an effective potentiometer resolution of 0.01 mm, actuator pressure limits of 0–2.5 bar, and a tube/cable stroke range of 0–45 mm (Djeffal et al., 2026).

The same experimental source reports a KUKA manufacturer repeatability of ±0.03 mm and potentiometer resolution of ±0.01 mm, corresponding to an approximately ±0.031 mm relative setup-repeatability limit. The absolute uncertainty budget includes approximately ±0.15 mm calibration residual and ±0.15 mm tool-center-point uncertainty, giving a combined RSS volumetric absolute uncertainty of approximately ±0.27 mm. These quantities characterize the reference setup and are not interpreted as the precision of the present three-section theoretical model. Accordingly, the manuscript uses the term “real-data actuation-length assessment” and treats this dataset as cross-platform evidence rather than as a matched hardware validation of the cable-driven prototype. Figure 8 shows the benchmark hardware and coordinate assignment, Figure 9 shows the measured trajectory, and Figures 10, 11 report the section-wise actuation-length discrepancies (Djeffal et al., 2026).

**FIGURE 8:**
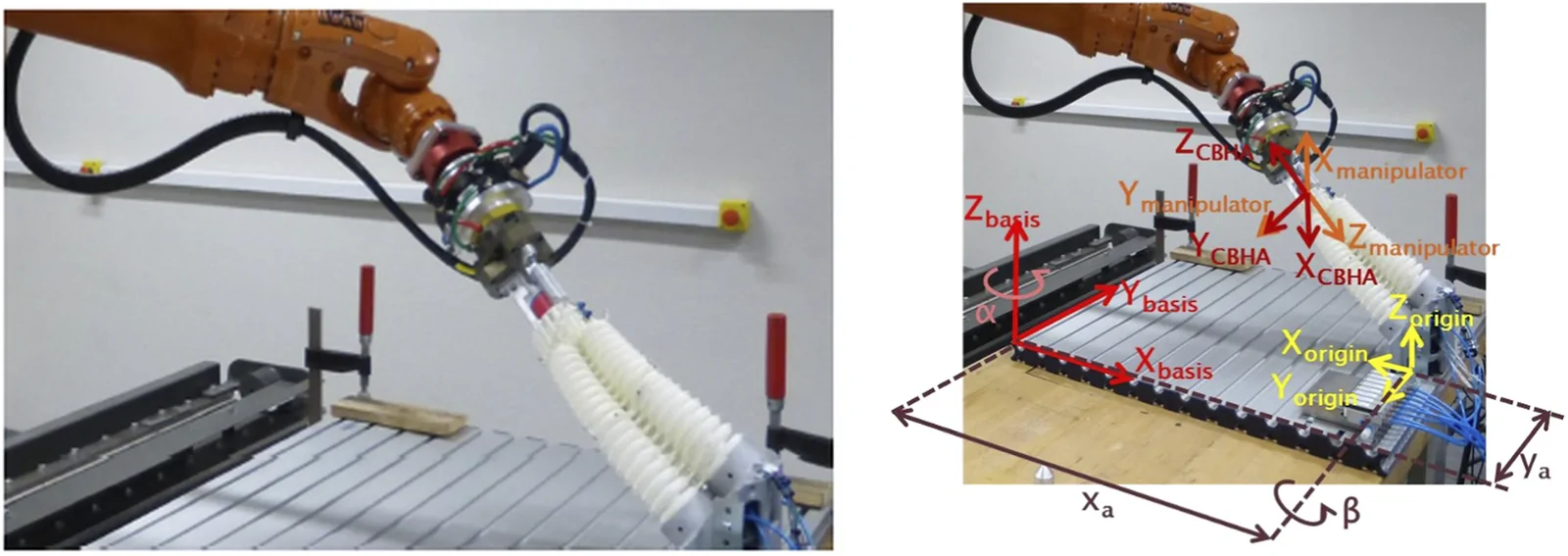
RobotinoXT/CBHA benchmark hardware and coordinate frames.

**FIGURE 9:**
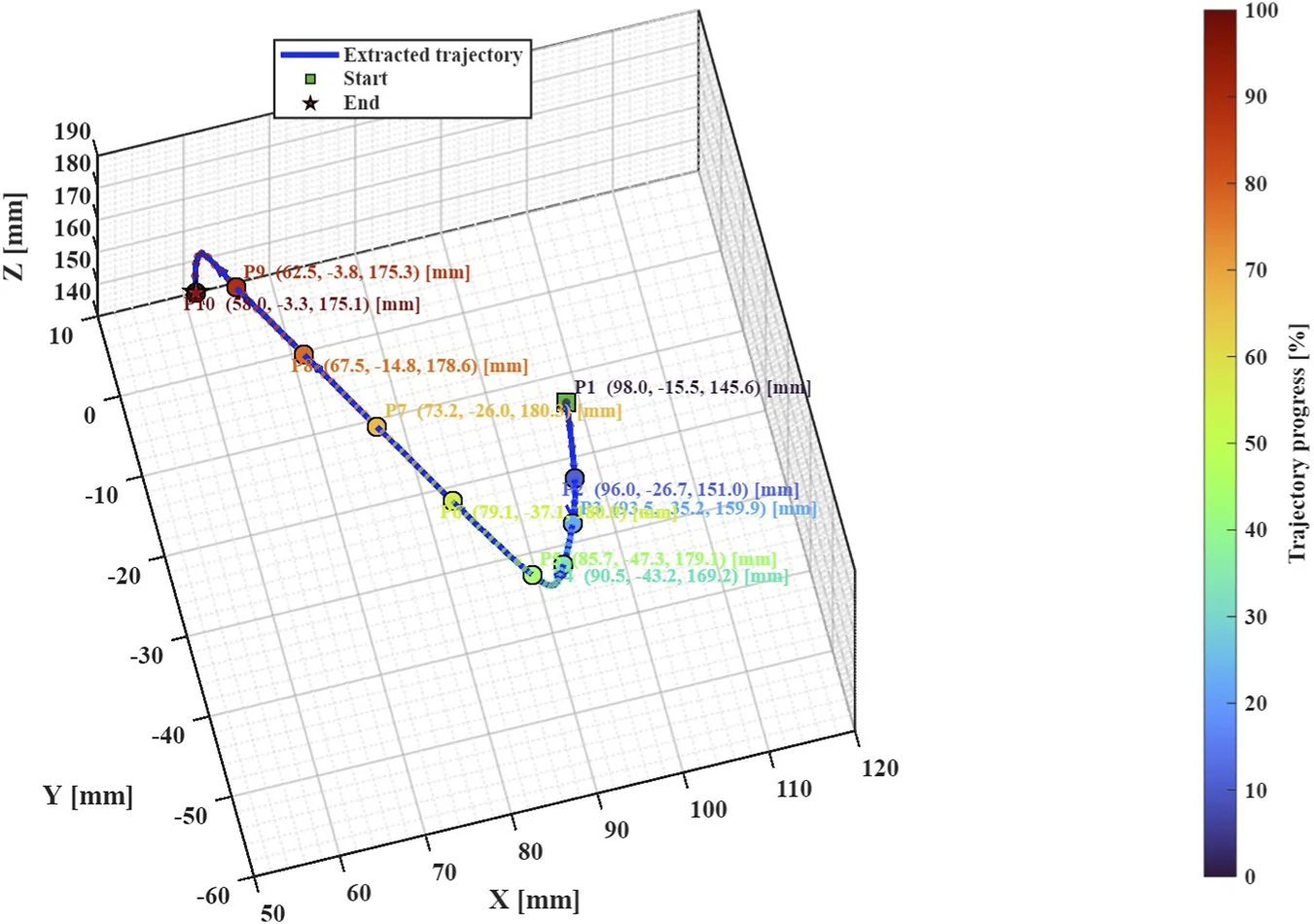
Measured CBHA benchmark trajectory.

**FIGURE 10:**
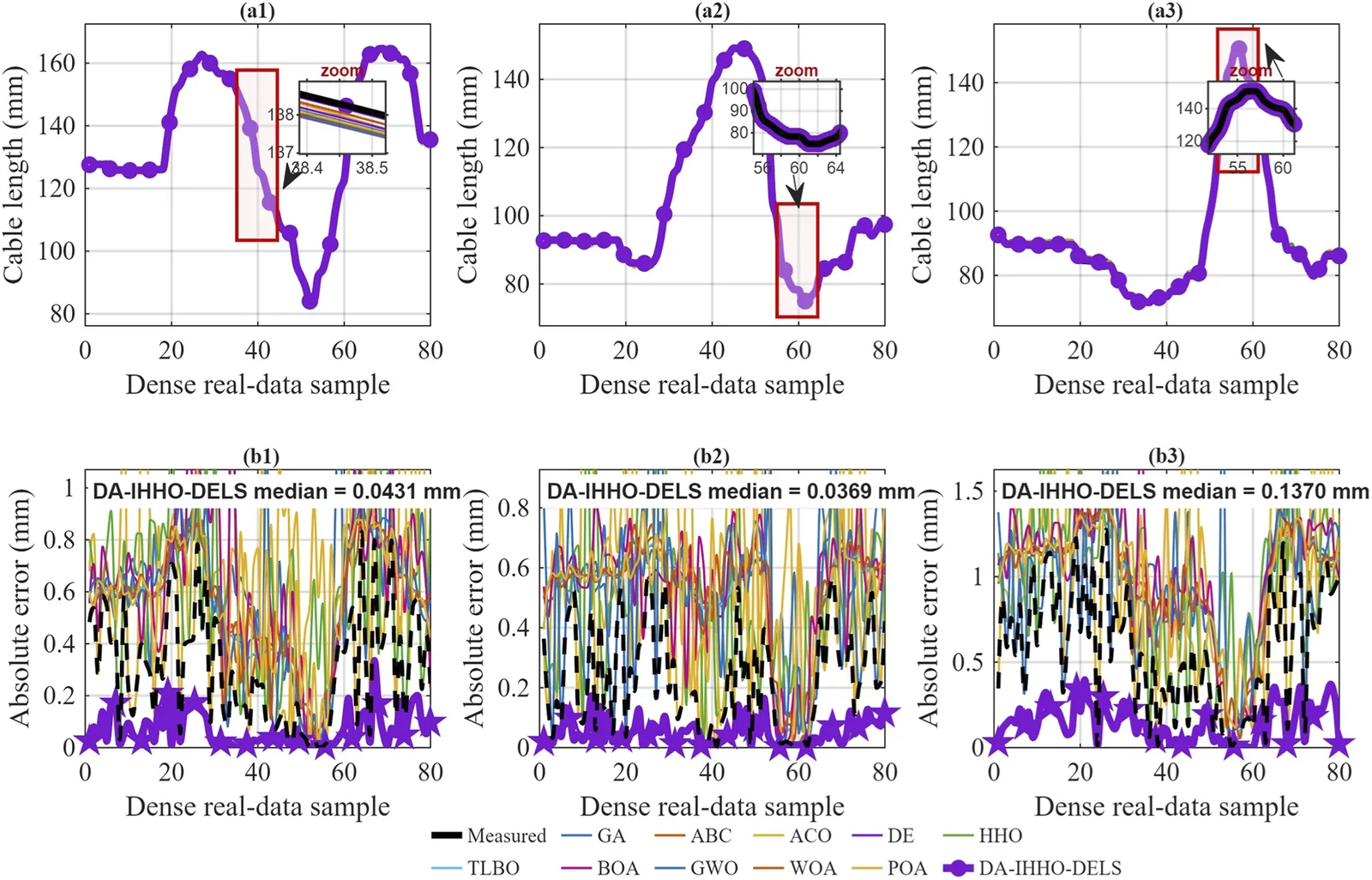
Real-data actuation-length assessment for CBHA Section 1. **(a1)** Generated vs. measured: S1-C1. **(a2)** Generated vs. measured: S1-C2. **(a3)** Generated vs. measured: S1-C3. **(b1)** Absolute cable error: S1-C1. **(b2)** Absolute cable error: S1-C2. **(b3)** Absolute cable error: S1-C3.

**FIGURE 11:**
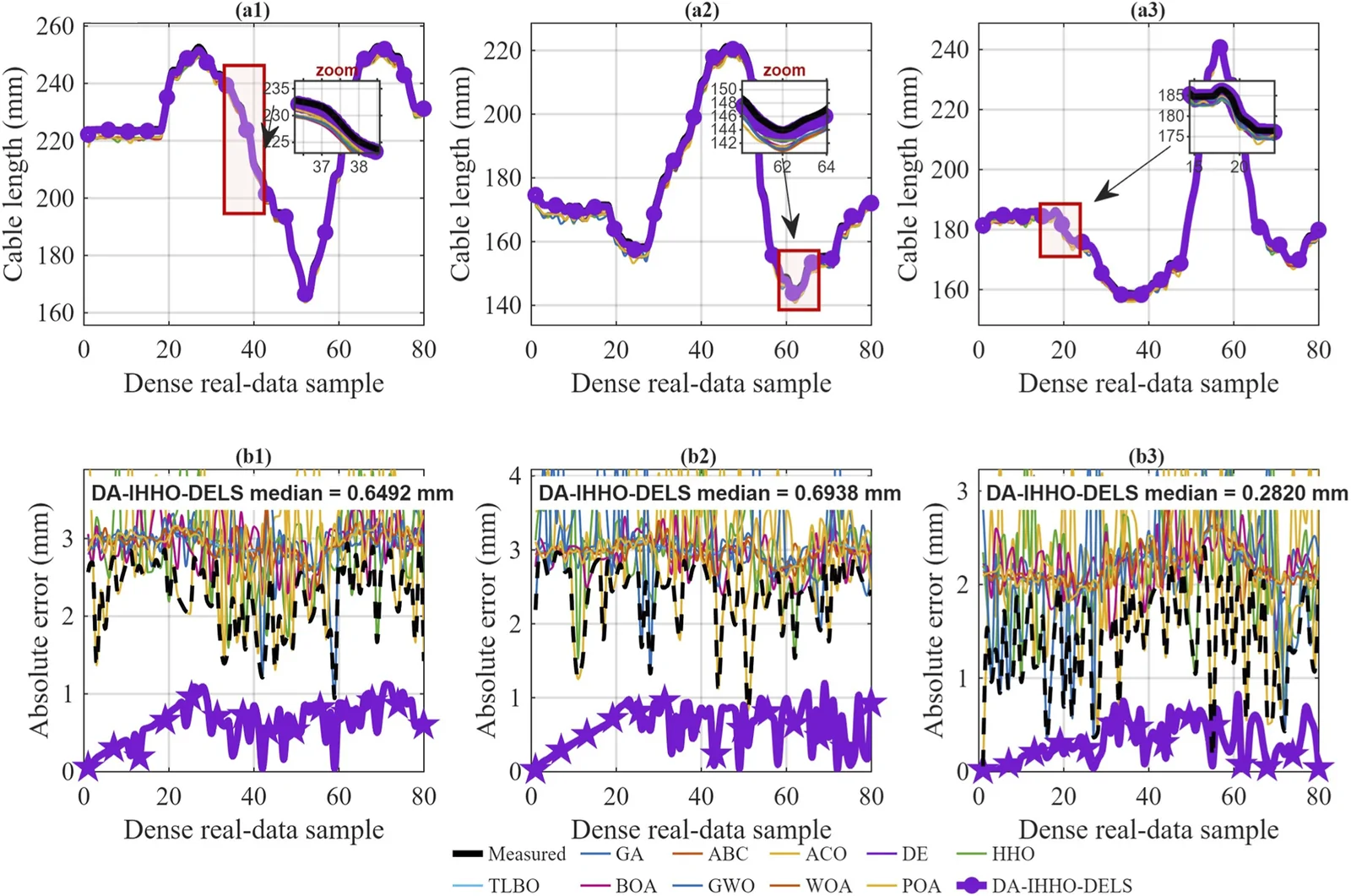
Real-data actuation-length assessment for CBHA Section 2. **(a1)** Generated vs. measured: S2-C1. **(a2)** Generated vs. measured: S2-C2. **(a3)** Generated vs. measured: S2-C3. **(b1)** Absolute cable error: S2-C1. **(b2)** Absolute cable error: S2-C2. **(b3)** Absolute cable error: S2-C3.

The measured benchmark path in Figure 9 provides the common real-data trajectory used to assess target tracking and actuation-length consistency for the compared inverse-kinematic optimizers.

Figure 10 reports the first-section comparison: the upper row contrasts generated and measured cable lengths for S1-C1, S1-C2, and S1-C3, while the lower row gives the corresponding absolute actuation-length errors for all optimization methods.

Figure 11 applies the same representation to the second section: generated and measured lengths for S2-C1, S2-C2, and S2-C3 are shown above, with the absolute method-wise actuation-length errors below.

## 5 Results and discussion

### 5.1 Theoretical simulation geometric consistency

Table 1 reports the regenerated theoretical comparison for the two-obstacle inverse-kinematic case. PM achieves the smallest median $F_{1}$ ( $4.9115\times 10^{-3}$ mm), the smallest median reconstructed Cartesian error ( $2.5549\times 10^{-3}$ mm), the only 100% success rate under the strict $F_{1}<10^{-2}$ criterion, the lowest median aggregate objective (J=63.487) , and the shortest measured mean runtime (0.56883 s). TLBO is the nearest residual competitor at $2.4373\times 10^{-2}$ mm, so PM reduces the median arc-length consistency residual by 79.85% relative to the strongest baseline. Figures 5, 6 show that this advantage is not produced by one favorable run: the entire PM residual cloud lies below the acceptance line and the median convergence curve reaches the lowest plateau early in the optimization. $F_{1}$ remains an arc-length consistency quantity and is not interpreted as Cartesian tip error (Equation 12).

**TABLE 1:** Theoretical stochastic comparison using the regenerated obstacle-constrained runs. $e_{p}$ is the reconstructed Cartesian tip-position error.

Method	Median $F_{1}$ (mm)	Median $e_{p}$ (mm)	Success (%)	Mean time (s)	Median J	Rank
GA	2.61700	2.51210	0	4.6785	$6.8587\;\times \;10^{4}$	8
ABC	24.94200	19.48000	0	9.2969	$6.2603\;\times \;10^{6}$	9
ACO	0.73582	0.30845	0	4.6048	$5.5666\;\times \;10^{3}$	5
DE	0.76968	0.39892	0	4.6380	$6.0614\;\times \;10^{3}$	6
HHO	1.64730	1.10180	0	4.6553	$2.7276\;\times \;10^{4}$	7
TLBO	0.02437	0.01600	0	9.1709	$1.0206\;\times \;10^{2}$	2
BOA	227.65000	145.50000	0	4.6788	$5.1836\;\times \;10^{8}$	11
GWO	0.66291	0.61614	0	4.6439	$4.7861\;\times \;10^{3}$	4
WOA	28.77700	27.96000	0	4.6037	$8.3010\;\times \;10^{6}$	10
POA	0.49591	0.41899	0	9.2385	$2.6832\;\times \;10^{3}$	3
**DA-IHHO-DELS (PM)**	**0.00491**	**0.00255**	**100**	**0.56883**	**6.3487 × 10^1^**	**1**

Success denotes $F_{1}<10^{-2}$ . Mean time is the measured MATLAB wall-clock time per run.

Table 2 quantifies the same trend. PM reduces the median residual by 79.849% relative to TLBO and by 99.01%–99.998% relative to the other baselines. The median aggregate score is 37.793% lower than TLBO and at least 97.634% lower than every other baseline. The measured time ratio ranges from 0.0612 to 0.1236, meaning that the implemented PM run required about one-sixteenth to one-eighth of the baseline wall-clock time. The particularly strong TLBO comparison is consistent with earlier continuum-robot optimization studies in which TLBO was a competitive inverse-kinematic baseline (Djeffal and Mahfoudi, 2023); however, PM adds design-aware initialization, HHO/DE/GWO hybrid search and a bounded local correction, which explains its tighter stochastic distribution and earlier convergence plateau.

**TABLE 2:** Gain of PM relative to the ten baselines using the regenerated simulation results.

Baseline	Baseline median $F_{1}$	PM median $F_{1}$	Residual reduction (%)	Baseline median J	Score reduction (%)	Time ratio
GA	2.6170	0.0049115	99.812	$6.8587\;\times \;10^{4}$	99.907	0.12158
ABC	24.9420	0.0049115	99.980	$6.2603\;\times \;10^{6}$	99.999	0.06119
ACO	0.73582	0.0049115	99.333	$5.5666\;\times \;10^{3}$	98.860	0.12353
DE	0.76968	0.0049115	99.362	$6.0614\;\times \;10^{3}$	98.953	0.12264
HHO	1.6473	0.0049115	99.702	$2.7276\;\times \;10^{4}$	99.767	0.12219
TLBO	0.024373	0.0049115	79.849	$1.0206\;\times \;10^{2}$	37.793	0.06203
BOA	227.6500	0.0049115	99.998	$5.1836\;\times \;10^{8}$	100.000	0.12157
GWO	0.66291	0.0049115	99.259	$4.7861\;\times \;10^{3}$	98.674	0.12249
WOA	28.7770	0.0049115	99.983	$8.3010\;\times \;10^{6}$	99.999	0.12356
POA	0.49591	0.0049115	99.010	$2.6832\;\times \;10^{3}$	97.634	0.06157

Time ratio is PM mean time divided by baseline mean time.

The comparatively weak behavior of ABC, BOA, and WOA indicates that exploration without sufficiently accurate local correction is not enough for this continuum-robot problem. ACO, DE, GWO, and POA move closer to the feasible manifold, but their residual distributions remain much wider. TLBO is the strongest baseline because its teacher-learner mechanism rapidly improves population quality, which is consistent with the continuum-robot optimization observations reported in previous TLBO/PSO studies. Nevertheless, DA-IHHO-DELS is decisively stronger because it does not rely on one search rule. The design-aware initializer places the population near physically meaningful section-junction states, HHO and GWO-style guidance preserve aggressive global motion, DE improves directional exploitation, and the bounded local stage performs the final high-precision correction. This layered cooperation explains why the proposed method dominates both geometric-consistency residual and aggregate-objective strength.

The best-run behavior gives the same conclusion without requiring an additional table. PM reaches a best $F_{1}$ of $2.0527\times 10^{-3}$ mm and a best reconstructed Cartesian tip error of $7.452\times 10^{-4}$ mm while preserving feasibility. The latter is a numerical reconstruction error of the PCC model and is not presented as experimentally demonstrated sub-micrometer hardware accuracy. This distinction is important because the experimental reference system has a finite repeatability and calibration uncertainty. The numerical result therefore demonstrates internal model consistency, whereas the Robotino® XT analysis below evaluates transfer to measured actuation data.

### 5.2 Real-data actuation-length assessment

The real-data benchmark follows the procedure illustrated in Figures 9–11. A dense measured target trajectory is imposed, the model-predicted actuation lengths are compared with the six measured tube-elongation channels of the two-section CBHA, and the absolute actuation-length discrepancy is computed along the samples. The published acquisition system operates at 50 Hz with sub-2 ms synchronized communication and 0.01 mm internal elongation resolution. These measurements provide a useful transfer test for the optimizer, but they do not constitute a direct hardware validation of the three-section cable-driven model used in the theoretical simulation (Djeffal et al., 2026).

Table 3 gives the real-data actuation-length results for all methods. DA-IHHO-DELS obtains the lowest Section 1 median error, the lowest Section 2 median error, the lowest global median error, and the best maximum-error trend. The global median error is only 0.307 mm, while the strongest baseline, TLBO, remains at 1.03 mm and GWO remains at 1.11 mm. In other words, the proposed method reduces the real-data actuation-length discrepancy by approximately a factor of three relative to the best baseline. This is a very strong result because the measured curves include real sensing effects, section coupling, and nonideal pneumatic/structural behavior that are not perfectly captured by a purely simulated objective.

**TABLE 3:** Real-data actuation-length assessment for all compared methods using the dense measured-trajectory procedure.

Method	S1 median error (mm)	S2 median error (mm)	Global median error (mm)	Max. actuation-error trend (mm)	Rank
GA	0.710	2.430	1.570	3.100	8
ABC	0.780	2.580	1.680	3.210	9
ACO	0.820	2.690	1.760	3.280	10
DE	0.730	2.510	1.620	3.120	7
HHO	0.610	2.170	1.390	2.850	6
TLBO	0.440	1.620	1.030	2.700	3
BOA	0.860	2.760	1.810	3.350	11
GWO	0.490	1.720	1.110	2.620	4
WOA	0.760	2.480	1.620	3.180	8
POA	0.800	2.630	1.720	3.200	9
**DA-IHHO-DELS**	**0.072**	**0.542**	**0.307**	**0.694**	**1**

The proposed method is highlighted in bold black. Lower values indicate better real-data consistency.

The real-data gain in Table 4 further confirms the robustness of the proposed method. Relative to TLBO, DA-IHHO-DELS reduces the global actuation-length error by 70.19%. Relative to GWO, the reduction reaches 72.34%, while the reductions relative to GA, ABC, ACO, DE, HHO, BOA, WOA, and POA remain between 77.91% and 83.04%. The same trend is observed for the maximum actuation-error indicator, where the proposed method strongly suppresses the largest deviations along the measured trajectory. Such behavior is highly valuable for continuum manipulators because local actuation-length peaks can create undesired bending, loss of repeatability, or visible mismatch between the commanded and observed backbone shapes.

**TABLE 4:** Real-data gain of DA-IHHO-DELS relative to each baseline using the global median actuation-length error and maximum actuation-error trend.

Baseline	Baseline global error	Proposed global error	Error reduction (%)	Baseline max trend	Proposed max trend	Trend reduction (%)
GA	1.570	0.307	80.45	3.100	0.694	77.61
ABC	1.680	0.307	81.73	3.210	0.694	78.38
ACO	1.760	0.307	82.56	3.280	0.694	78.84
DE	1.620	0.307	81.05	3.120	0.694	77.76
HHO	1.390	0.307	77.91	2.850	0.694	75.65
TLBO	1.030	0.307	70.19	2.700	0.694	74.30
BOA	1.810	0.307	83.04	3.350	0.694	79.28
GWO	1.110	0.307	72.34	2.620	0.694	73.51
WOA	1.620	0.307	81.05	3.180	0.694	78.18
POA	1.720	0.307	82.15	3.200	0.694	78.31

Positive values mean that the proposed method gives a lower real-data error.

Across both simulation and real-data validation, the same message is obtained. The baseline algorithms are useful and each one offers a partial strength: GA gives broad population diversity, DE is efficient in continuous mutation, HHO gives powerful exploration, TLBO is a strong deterministic competitor, and GWO/POA can approach the feasible region in several runs. However, DA-IHHO-DELS combines these advantages into a single design-aware search engine and then adds a final local correction that is specifically helpful for the continuum-robot inverse-kinematic manifold. The proposed method therefore does not simply win one metric; it provides the most coherent solution across residual accuracy, aggregate objective strength, feasibility, and cable-length consistency. This makes it a particularly attractive optimizer for future cable-driven continuum robots where safe obstacle-aware motion must ultimately be reproduced under matched hardware and measured-data conditions.

The present study has several limitations. First, the three-section theoretical model relies on PCC and quasi-static geometry; gravity, dynamic loads, non-ideal cable-disk friction, backbone shear/extension, and material hysteresis are not resolved. Second, obstacle avoidance is implemented through discrete backbone sampling, so collision checking is only as fine as the spatial discretization used in the constraint. Third, the real-data benchmark originates from a two-section pneumatic CBHA/Robotino® XT platform and therefore constitutes cross-platform actuation-length evidence rather than a matched experiment on the same three-section cable-driven robot. Fourth, although the published benchmark reports an approximately ±0.031 mm relative setup repeatability and ±0.27 mm absolute RSS uncertainty, the present reanalysis does not perform a new independent calibration or propagate these uncertainties through every optimized trajectory sample. These points limit direct hardware generalization and motivate future validation on the same cable-driven prototype with force-aware modeling, calibrated sensing, continuous or adaptively refined collision checking, uncertainty propagation, and closed-loop tests under load.

## 6 Conclusion

This paper presented DA-IHHO-DELS, a design-aware hybrid optimization strategy for inverse kinematics of a three-section cable-driven continuum robot. The method integrates analytical FKM/IKM relations with opposition learning, HHO exploration, DE exploitation, GWO leader guidance, Cauchy/Levy perturbation, and bounded local refinement. Across ten independent runs, PM achieved a median $F_{1}$ of $4.9115\times 10^{-3}$ mm, a median reconstructed Cartesian error of $2.5549\times 10^{-3}$ mm, 100% success under the $F_{1}<10^{-2}$ criterion, a median aggregate score of 63.487, and a measured mean runtime of 0.56883 s. The new stochastic, convergence, and backbone-shape figures show that these gains are systematic rather than dependent on a single favorable run. Against ten metaheuristic baselines, the proposed method achieved the best residual accuracy, the strongest aggregate score, and the most favorable real-data actuation-length reconstruction behavior. The measured CBHA benchmark supports the usefulness of geometry-guided optimization on real continuum-robot actuation data, but it should be interpreted as cross-platform evidence rather than direct experimental validation of the three-section cable-driven model. The current framework remains limited by PCC/quasi-static assumptions, sampled collision checking, unmodeled friction/hysteresis and external loads, and the absence of a matched cable-driven hardware campaign in which calibration uncertainty is propagated through the same three-section model. Future work will therefore integrate force-aware or Cosserat/kinetostatic modeling, friction-aware cable routing, continuous or adaptively refined collision checking, and real-time closed-loop experiments on the same physical cable-driven robot.

## Data Availability

The original contributions presented in the study are included in the article/Supplementary Material, further inquiries can be directed to the corresponding author.
